# Assessment of Sleep Quality and Modifiable Disruptors in Postoperative Paediatric Patients: A Clinical Audit

**DOI:** 10.7759/cureus.118296

**Published:** 2026-10-21

**Authors:** Zeenia Merali, Mohammed Hazara

**Affiliations:** 1 Paediatrics and Child Health, Royal National Orthopaedic Hospital NHS Trust, Watford, GBR; 2 Internal Medicine, University Hospital Southampton NHS Foundation Trust, Southampton, GBR

**Keywords:** factors affecting sleep quality, paediatric sleep problems, pediatric sleep questionnaire (psq), postoperative sleep disturbance, postoperative sleep quality, quality of sleep, sleep assessment, sleep problems

## Abstract

Background

Sleep disruption is common during postoperative hospitalisation and may be influenced by pain, overnight clinical care, and environmental factors. This quality improvement project assessed sleep quality among postoperative paediatric inpatients and identified potentially modifiable contributors to sleep disturbance.

Methodology

A clinical audit was conducted on a paediatric inpatient ward between April and June 2026. Children aged 0-18 years admitted following elective or emergency orthopaedic surgery and requiring at least one overnight postoperative stay were eligible. Parents or carers completed a questionnaire after the child’s first postoperative night assessing sleep quality, nighttime awakenings, causes of sleep disturbance, ward noise and lighting, clustering of overnight care, assessment of sleep needs, and parent/carer sleep. Responses were analysed descriptively using frequencies and percentages.

Results

In total, 19 questionnaires were included: 14 from children accommodated in multi-bed bays and five from children in individual cubicles. In bays, eight of 14 (57.1%) children were reported to have fair sleep and six (42.9%) woke more than five times overnight. In cubicles, two of five (40.0%) children were reported to have good sleep and one (20.0%) woke more than five times. Nursing observations were reported as a contributor to sleep disturbance in 10 of 14 (71.4%) children in bays and four of five (80.0%) in cubicles. Pain contributed to sleep disturbance in 10 of 19 (52.6%) children overall. Most respondents reported that the ward was quiet overnight and that lights were kept low. However, only seven of 19 (36.8%) families reported being asked about their child’s sleep needs. Parent/carer sleep was also frequently disrupted.

Conclusions

Postoperative sleep disruption was common, with nursing observations and pain among the most frequently reported contributors. Despite generally favourable perceptions of overnight noise and lighting, opportunities for improvement were identified in assessment of individual sleep needs, coordination of overnight care where clinically appropriate, and optimisation of pain management. A subsequent audit cycle following implementation of these measures will assess their impact on sleep-related outcomes.

## Introduction

Sleep is an important component of recovery, particularly in postoperative patients. Although sleep complaints are common during hospitalisation, there is limited evidence examining the effects of hospitalisation on sleep in paediatric patients and their parents or carers [1]. Factors contributing to sleep disturbance are often modifiable and include nursing interventions, environmental noise, lighting, pain, and unfamiliar surroundings. Studies have demonstrated increased nighttime awakenings and disruption of normal sleep in hospitalised children compared with sleep at home [1,2].

The postoperative period may present additional challenges to maintaining adequate sleep. Pain and discomfort following surgery can interfere with sleep, while frequent clinical observations and nursing interventions may contribute to repeated overnight awakenings. Studies suggest that sleep disturbances are associated with exacerbations of pain and may influence the pain experience [3]. Furthermore, sleep-wake disruption in hospitalised patients can have negative physical, emotional, and cognitive effects, which may subsequently affect the overall hospital experience and length of stay [4]. While some overnight interruptions are essential for appropriate clinical management and patient safety, several potentially modifiable aspects of inpatient care may provide opportunities to reduce unnecessary sleep disruption.

Previous research has identified noise, the number of patients sharing a room, pain, and anxiety among the most frequent modifiable factors affecting sleep in hospitalised children. Hospitalised children have also been reported to sleep approximately 0.7-3.8 hours less than recommended for their age [5].

Objective assessment using actigraphy has similarly demonstrated short and fragmented sleep during paediatric hospitalisation, with frequent nighttime awakenings across different age groups [6]. Nursing care is an important contributor to this disruption; a review specifically examining overnight vital sign monitoring found that vital sign measurements were consistently among the most common causes of sleep interruption in hospitalised children [7].

Sleep disturbance may be particularly relevant following surgery. In children undergoing major surgery, postoperative sleep quality and sleep efficiency have been shown to decline following surgery, with poorer nighttime sleep quality associated with greater pain the following day [8]. In another study of children undergoing inpatient surgery, higher postoperative pain scores were associated with reduced objectively measured sleep time [9]. These findings highlight the importance of identifying potentially modifiable causes of sleep disturbance and developing interventions aimed at improving sleep during hospitalisation.

This quality improvement project aimed primarily to assess sleep quality among postoperative paediatric patients following orthopaedic surgery. Secondary objectives were to identify reported factors contributing to sleep disturbance, assess the overnight ward environment and sleep-related care practices, including whether individual sleep needs had been discussed, and describe sleep quality among accompanying parents or carers. The findings were intended to identify potentially modifiable aspects of overnight care and inform future interventions to promote a more sleep-supportive environment for postoperative paediatric patients and their families.

## Materials and methods

Study design and setting

This study was conducted in a pediatric inpatient ward between April and June 2026 and aimed to assess the quality of sleep in postoperative patients and identify the modifiable risk factors potentiating sleep disturbance. The experience of both patients and their carers was assessed. In the hospital, patients are accommodated in both bays, which contain multiple beds, or individual cubicles. Patients in both were included in the assessment.

Participants included children aged 0-18 years admitted to the paediatric ward following an elective or emergency orthopaedic surgery who had stayed in the ward for at least one night. To complete the questionnaire, a carer was required to be present, but responses could be provided by the child or carer. Patients undergoing day-case surgeries, on continuous sedation, or mechanical ventilation, and those whose carer was unable to complete the questionnaire were excluded.

A total of 19 completed questionnaires were included. From these, 14 were related to patients staying in the bays and five were related to patients staying in the cubicles. As cubicles are mostly reserved for patients who could not complete the questionnaire, this resulted in a lower number of questionnaires from this cohort.

Data collection

Eligible patients and their carers were approached in person by a doctor following the first postoperative night. The questionnaire and purpose of the study were explained, and consent was obtained to administer the questionnaire. The questionnaire used was not piloted or validated for this audit.

For children who were too young or unable to verbally communicate their sleep experience, the questionnaire was filled out by the accompanying carer. The questionnaire was divided into four sections. The first section aimed to assess the child’s sleep, including potential factors that disrupted sleep, pain control and an assessment that compared sleep at home versus postoperative sleep in the ward, as well as electronic device usage before sleep. The second part of the questionnaire aimed to assess the carers’ sleep in relation to modifiable factors that cause a disturbance. The third section aimed to assess the ward environment and care, including lighting, noise, nursing interventions, as well as identification of sleep needs for the child. The last section was a free text for either the carer or patient to indicate what they thought would most improve sleep in the ward.

Standards and outcome measures

The audit assessed practice against four predetermined standards informed by published guidance and evidence relating to sleep during hospitalisation.

Standard 1 stated that children should be provided with an environment that promotes rest and sleep through the minimisation of noise, light, and avoidable overnight interruptions. This was informed by National Institute for Health and Care Excellence (NICE) guidance recommending a calm inpatient environment with minimal background noise, particularly at night [10].

Standard 2 stated that overnight care activities should be clustered where clinically appropriate to reduce avoidable sleep disruption. This was informed by published evidence identifying overnight vital sign monitoring and nursing care as contributors to sleep interruption in hospitalised children [7].

Standard 3 stated that parents and carers should feel able to raise concerns about their child’s sleep with staff, reflecting NICE guidance on communication and involvement of children and families in healthcare [10].

Standard 4 stated that nighttime environmental noise should be minimised, with World Health Organization (WHO) recommendations of an average indoor sound level below 30 dB and maximum noise events below 40 dB used as environmental benchmarks [11].

These standards were used to guide the descriptive assessment of existing ward practices. The questionnaire assessed whether families reported that their child’s individual sleep needs had been discussed during admission, which was considered a related measure under Standard 3 rather than a direct assessment of whether families felt able to raise concerns. No numerical percentage compliance targets had been specified in the original audit documentation. Objective environmental noise measurements were not undertaken; therefore, compliance with WHO noise thresholds could not be directly assessed.

NICE guidance on the experiences of children and young people using healthcare services was also considered in relation to providing a calm environment and minimising disturbance from background noise to support rest and sleep [10].

The principal outcome measures were the child’s sleep quality, frequency of nighttime awakenings, reported causes of sleep disturbance, environmental factors affecting sleep, whether overnight care activities were clustered where clinically appropriate, and whether the child’s sleep needs had been discussed with the parent or carer.

The questionnaire assessed whether parents or carers reported that their child’s individual sleep needs had been discussed during admission. This was considered a related measure of sleep-related communication under Standard 3. However, it did not directly assess whether parents or carers felt comfortable raising concerns about their child’s sleep with staff and therefore could not establish compliance with this standard.

Parent/Carer-reported sleep quality was assessed as an additional outcome. The questionnaire findings were summarised descriptively. No numerical percentage compliance targets had been specified for the four standards in the original audit documentation. Furthermore, objective sound measurements were not collected; therefore, the WHO noise thresholds were used as guidance benchmarks rather than directly measured compliance criteria. The same outcomes were assessed, where applicable, among children staying in bays and those staying in cubicles.

Data analysis

The questionnaires were compiled, tabulated, and analysed using percentages and descriptive statistics. The results were then summarised according to patients staying in bays versus cubicles. Regarding the causes of sleep disturbances, patients were allowed to pick more than one response. Due to the small sample size, the analyses were descriptive with no statistical comparisons made between the groups of data.

Questionnaire development and data collection

A structured questionnaire was developed by the authors specifically for this clinical audit to assess sleep during the first postoperative night. The questionnaire was not adapted from, and did not reproduce, a previously published questionnaire, validated scale, or proprietary assessment tool. Therefore, no external licence or permission was required for its use. The complete questionnaire is provided in the Appendices.

Ethics and governance

This project was conducted as a clinical audit and quality improvement initiative and was registered through the hospital’s local clinical audit/governance process. As the project evaluated existing clinical practice and did not involve an alteration to patients’ clinical care or allocation to an experimental intervention, formal research ethics committee approval was not required. Participation in the questionnaire was voluntary, and consent to participate was included within the audit eligibility criteria. Data were collected anonymously, with no names, NHS numbers, or other directly identifiable patient information recorded. Data were analysed and reported in aggregate to maintain patient and family confidentiality.

## Results

Nighttime awakenings

A total of 19 questionnaires were included in the analysis. Of these, 14 (73.7%) were from children sleeping in the multi-bed bays and five (26.3%) were from children staying in the cubicles.

From the subset of children staying in the bays, sleep quality rated by the parent/carer was most commonly rated as fair, seen in eight out of the 14 (57.1%) responses; three (21.4%) were rated as good, two (14.3%) as poor, and only one (7.1%) response was rated as very good, with no child having rated very poor sleep. Among the children staying in the individual cubicles, two out of five (40%) had good sleep, and the remaining three children were each rated as fair (20.0%), poor (20.0%) and very poor (20.0%).

Nighttime awakenings were common, with six out of the 14 (42.9%) children staying in the bays indicating waking up more than five times overnight, four (28.6%) children from the same cohort woke up three to four times overnight, and four (28.6%) children woke up one to two times. In the cubicles, three out of five (60.0%) children woke up one to two times, one (20.0%) child woke up three to four times, and one (20.0%) child woke up five or more times. Postoperative sleep quality and nighttime awakenings according to accommodation type are summarised in Table 1.

**Table 1:** Child sleep quality and nighttime awakenings reported by the children and/or carers.

Outcome	Bay (n = 14)	Cubicle (n = 5)
Sleep quality: Very poor	0 (0.0%)	1 (20.0%)
Sleep quality: Poor	2 (14.3%)	1 (20.0%)
Sleep quality: Fair	8 (57.1%)	1 (20.0%)
Sleep quality: Good	3 (21.4%)	2 (40.0%)
Sleep quality: Very good	1 (7.1%)	0 (0.0%)
Nighttime awakenings: 1–2	4 (28.6%)	3 (60.0%)
Nighttime awakenings: 3–4	4 (28.6%)	1 (20.0%)
Nighttime awakenings: ≥5	6 (42.9%)	1 (20.0%)

Factors contributing to postoperative sleep disturbance

Among children accommodated in bays, nursing observations were the most frequently reported factor, identified in 10 of 14 (71.4%) responses, followed by pain in nine (64.3%). Medication timing and other factors were each reported in four (28.6%) responses, while ward noise, positioning, and brace or cast were each reported in three (21.4%), and lighting in one (7.1%). Among children accommodated in cubicles, nursing observations were reported in four of five (80.0%) responses. Pain, ward noise, lighting, positioning, brace or cast, and medication timing were each reported once (20.0%). Washroom-related disturbance was recorded twice in the free-text field. As participants could select more than one factor, percentages do not total 100%. The reported factors contributing to postoperative sleep disturbance are summarised in Table 2.

**Table 2:** Parent/Carer-reported factors contributing to postoperative sleep disturbance.

Factors contributing to sleep disturbance	Bay (n = 14)	Cubicle (n = 5)
Nursing observations	10 (71.4%)	4 (80.0%)
Pain	9 (64.3%)	1 (20.0%)
Medication timing	4 (28.6%)	1 (20.0%)
Ward noise	3 (21.4%)	1 (20.0%)
Positioning/Brace/Cast	3 (21.4%)	1 (20.0%)
Lighting	1 (7.1%)	1 (20.0%)
Other	4 (28.6%)	2 (40.0%)

Ward environment and overnight care

Responses for this section were largely positive, with 13 recorded responses from children residing in the bays regarding ward noise. From this, 11 (84.6%) reported that the ward was quiet overnight, while two (15.4%) rated the ward ‘somewhat’ quiet. From the five participants in the cubicles, four (80.0%) reported the ward quiet and one (20.0%) rated the ward ‘somewhat’ quiet.

For overnight lighting, 12 out of the 14 (85.7%) responses in bays agreed or strongly agreed that lights were kept low, compared to a response of 100% of responses in the cubicles.

Regarding clustering of overnight care, there were a total of 13 responses in the bay group. Nine of these (69.2%) reported clustering of care, two (15.4%) reported ‘sometimes’, one (7.7%) reported ‘no’, and one responder was ‘unsure’ (7.7%).

When inquired about their child’s sleep needs, five of the 14 (35.7%) respondents in bays and two out of the five (40.0%) in cubicles reported that they had been asked about their child’s sleep needs. In total, seven out of the 19 (36.8%) families reported that their child’s sleep needs had been discussed during their admission. Responses relating to the overnight ward environment and sleep-related care practices are summarised in Table 3. Denominators vary because not all respondents answered every questionnaire item.

**Table 3:** Overnight ward environment and sleep-related care practices.

Outcome	Bay (n = 14)	Cubicle (n = 5)
Ward quiet overnight: Yes	11/13 (84.6%)	4/5 (80.0%)
Lights kept low: Agree/strongly agree	12/14 (85.7%)	5/5 (100.0%)
Overnight care clustered: Yes	9/13 (69.2%)	2/4 (50.0%)
Family asked about child’s sleep needs: Yes	5/14 (35.7%)	2/5 (40.0%)

Parent/Carer sleep

This section was filled out by the parents/carers staying overnight with the child. Among the parents staying with the child in the bays, there were 13 recorded responses regarding sleep quality. Seven (53.8%) rated their sleep as fair, four (30.8%) as poor, and two (15.4%) as good. Among the five carers in the cubicles, three (60.0%) had poor sleep, one (20.0%) had fair sleep, and one (20.0%) had very poor sleep.

The carers also had the option of identifying multiple factors that contributed to their sleep disturbance. From the bays, six responses indicated staff-related interruptions, five responses indicated child waking, four ticked noise, three identified anxiety or worry, and one each specified chair/bed discomfort. The last three additional responses recorded ‘other’ as a factor.

From the cubicle group, staff-related interruptions and chair/bed discomfort were each recorded four times, child waking was recorded three times, while noise, lighting and anxiety/worry were each reported twice. In the free-text section, other factors identified were feeling cold and air conditioning. Parent/carer-reported sleep quality according to the child’s accommodation type is summarised in Table 4.

**Table 4:** Parent/Carer-reported sleep quality.

Parent/Carer sleep quality	Bay (n = 13)	Cubicle (n = 5)
Very poor	0 (0.0%)	1 (20.0%)
Poor	4 (30.8%)	3 (60.0%)
Fair	7 (53.8%)	1 (20.0%)
Good	2 (15.4%)	0 (0.0%)
Very good	0 (0.0%)	0 (0.0%)

## Discussion

This audit demonstrated that sleep disruption was common among postoperative paediatric inpatients, with frequent nighttime awakenings reported. Nursing observations and pain were the most commonly reported contributors to sleep disturbance. Although perceptions of the overnight ward environment were generally favourable, particularly regarding noise and lighting, opportunities for improvement were identified in the assessment of individual sleep needs and the organisation of overnight care. Sleep disruption was also common among accompanying parents and carers.

These findings are consistent with previous literature demonstrating that hospitalisation can disrupt normal sleep in children. Meltzer et al. identified noise, worry, pain, and vital sign checks as factors associated with sleep disruption in hospitalised children and their parents [1]. Similarly, a scoping review of hospitalised children and adolescents identified predominantly modifiable external factors, including nursing care activities and noise from equipment and other patients [2]. More specifically, overnight vital sign monitoring has been identified as an important contributor to sleep disruption in hospitalised children [7]. As seen in the present audit, nursing observations were the most frequently reported contributor to sleep disturbance in both bays and cubicles. Clinical observations remain essential for patient safety, particularly in the postoperative period; however, where clinically appropriate, coordinating observations with medication administration and other nursing activities may reduce the number of separate overnight interruptions.

Pain was another important contributor to sleep disturbance. Pain was reported as a cause of sleep disruption in nine of 14 (64.3%) children accommodated in bays and one of five (20.0%) children accommodated in cubicles, representing 10 of 19 (52.6%) children overall. The relationship between sleep and pain is complex, with evidence suggesting that sleep disturbance may influence the pain experience [3]. This relationship has also been demonstrated in paediatric surgical populations. Poorer nighttime sleep quality following major paediatric surgery has been associated with greater pain intensity the following day [8], while higher postoperative pain scores have also been associated with reduced objectively measured sleep among children undergoing inpatient surgery [9]. These findings, together with the present audit, support continued attention to overnight pain assessment and management, including timely administration of analgesia and assessment for breakthrough pain.

Perceptions of the overnight ward environment were generally favourable. Among respondents accommodated in bays, 11 of 13 (84.6%) reported that the ward was quiet overnight, while four of five (80.0%) respondents in cubicles reported the same. Similarly, 12 of 14 (85.7%) respondents in bays and all five (100%) respondents in cubicles agreed or strongly agreed that lights were kept low at night. Nevertheless, sleep disruption remained common. Nursing observations were reported as a cause of sleep disturbance in 10 of 14 (71.4%) children in bays and four of five (80.0%) children in cubicles, whereas ward noise was reported by three of 14 (21.4%) and one of five (20.0%), respectively. Lighting was reported as a source of sleep disturbance by only one child in each group (7.1% and 20.0%, respectively).

These findings suggest that maintaining a quiet, low-light environment alone may not be sufficient to promote uninterrupted sleep in postoperative patients and that care-related and patient-related factors should also be considered. Nevertheless, environmental factors remain relevant, with objective monitoring in hospitalised children demonstrating associations between sound and light events and nighttime waking [6]. NICE guidance recommends that paediatric inpatient environments should have as little disturbance from background noise as possible and should be sufficiently quiet for rest and sleep, particularly at night [10]. WHO guidance also recognises environmental noise as an important contributor to sleep disturbance and provides guidance regarding noise in hospital environments [11]. However, environmental noise and lighting in the present audit were assessed through parent/carer perceptions rather than objective measurements. Therefore, it was not possible to determine objectively whether recommended environmental conditions were achieved.

Differences in sleep outcomes were observed between children accommodated in bays and cubicles. More than five nighttime awakenings were reported in six of 14 (42.9%) children in bays, compared with one of five (20.0%) children in cubicles. Previous objective research has also suggested that room type may influence sleep, with shorter total nighttime sleep reported among children sharing rooms compared with those accommodated in private rooms [6]. However, the findings of the present audit should be interpreted cautiously because only five children accommodated in cubicles were included, compared with 14 in bays. The audit was not designed or powered to determine whether room type independently influenced sleep quality, and other factors such as age, type of surgery, pain, clinical condition, or monitoring requirements may have contributed to the observed differences. The findings should therefore be regarded as descriptive rather than evidence that one type of accommodation provides better sleep than another.

A particularly modifiable finding was the limited assessment of children’s individual sleep needs. As only seven of 19 (36.8%) families reported having been asked about their child’s sleep needs, incorporating a brief assessment of usual sleep routines into preoperative assessment, admission, or postoperative care could provide a simple opportunity to individualise overnight care. This is consistent with qualitative research highlighting the importance of coordination of care and the role of parents in advocating for their child’s sleep during hospitalisation [12]. Although this finding does not directly measure whether families felt able to raise sleep-related concerns, it suggests an opportunity to strengthen routine discussions about sleep needs and preferences during inpatient care, such as usual bedtime, environmental conditions, and care routines that could be accommodated where clinically appropriate.

Sleep disruption was also reported among accompanying parents and carers. Among parents/carers of children accommodated in bays, 11 of 13 (84.6%) rated their sleep as fair or poor, while all five parents/carers of children in cubicles rated their sleep as fair, poor, or very poor. Staff-related interruptions and the child waking were commonly reported contributors to disturbed parent/carer sleep. This is consistent with previous research demonstrating that parents staying overnight with hospitalised children may also experience disrupted sleep [1]. A systematic integrative review similarly identified impaired sleep among parents staying overnight with their hospitalised children, with psychological, environmental, and social factors contributing to sleep disturbance [13]. Although parent/carer sleep was a secondary outcome of this audit, reducing avoidable overnight interruptions and improving the sleep environment may have benefits for both children and their accompanying carers, while ensuring that necessary clinical care and patient safety remain the priority.

The findings identified several practical and potentially modifiable targets for improving sleep during postoperative care. Nursing observations were reported as a cause of sleep disturbance in 10 of 14 (71.4%) children in bays and four of five (80.0%) children in cubicles, supporting the consideration of clustering observations, medication administration, and other overnight care activities where clinically appropriate. Pain was also reported as contributing to sleep disturbance in 10 of 19 (52.6%) children overall, highlighting the importance of ongoing assessment and optimisation of overnight pain management. In addition, only seven of 19 (36.8%) families reported being asked about their child’s sleep needs, identifying routine assessment of individual sleep needs as a simple and feasible target for improvement. As most respondents reported favourable perceptions of overnight noise and lighting, existing measures to maintain a quiet, low-light environment should be continued alongside interventions addressing care-related interruptions and pain.

Evidence from paediatric inpatient settings suggests that targeted sleep-promoting interventions can improve these outcomes. An educational intervention incorporating evidence-based sleep-promoting practices was associated with fewer nighttime awakenings, including a reduction in awakenings attributed to nursing care [14]. This supports the use of a multifaceted quality improvement approach rather than focusing solely on environmental noise or lighting. Following implementation of these measures, a subsequent audit cycle should reassess the same key outcomes to determine whether sleep quality and sleep-related care have improved.

Limitations

This audit has several limitations. First, it was conducted at a single specialist paediatric orthopaedic centre and included a small sample of 19 completed questionnaires, with unequal representation between children accommodated in bays (n = 14) and cubicles (n = 5). These factors limit the generalisability of the findings, and comparisons between accommodation types should be interpreted cautiously. The analysis was descriptive, and no inferential statistical comparisons were performed.

Second, the number of eligible families approached, refusals, and exclusions was not systematically recorded, preventing calculation of an accurate response rate and limiting assessment of potential non-response bias. Obtaining responses from cubicles was more challenging because these rooms generally accommodated more clinically vulnerable patients, which may have contributed to their smaller representation. Furthermore, the clinical criteria for allocation to bays or cubicles were not documented as part of the audit, limiting assessment of potential selection bias and comparability between groups.

Third, the questionnaire was developed specifically for this audit and was neither formally validated nor piloted before data collection. Sleep quality was assessed using subjective reports from children and/or their parents or carers. Although many questionnaires were completed by parents or carers, some children provided responses directly, and respondent identity was not recorded for individual questionnaires. Parent/Carer proxy reporting may introduce bias, as parental perceptions of their child’s sleep may be influenced by their own sleep experiences and may not capture awakenings that do not disturb the parent or carer. Additionally, sleep was assessed following only the first postoperative night, preventing evaluation of changes in sleep quality over subsequent nights.

Fourth, demographic and clinical characteristics, including patient age, surgical procedure type and complexity, analgesic requirements and overnight monitoring needs, were not available in the audit dataset. Baseline sleep patterns at home were also not included in the reported analysis. These factors may influence postoperative sleep quality, and their absence limited assessment of potential confounding and comparability between patients accommodated in bays and cubicles.

Fifth, some questionnaire items had missing responses, resulting in varying denominators and potentially affecting the completeness of the findings. The audit relied on subjective reports of sleep quality and environmental disturbances rather than objective sleep monitoring, such as actigraphy, or environmental measurements using sound-level and light meters. Consequently, reported contributors to sleep disturbance represent perceived associations rather than established causal factors, and compliance with specific environmental thresholds could not be directly assessed. Furthermore, numerical percentage compliance targets were not specified for the predetermined audit standards, limiting formal assessment of adherence to these standards.

Finally, this audit represents the baseline assessment phase (Cycle 1) of a quality improvement project. Although the findings identified potentially modifiable aspects of overnight care, no interventions or subsequent re-audit (Cycle 2) were undertaken within the reported project. Therefore, the effectiveness of the proposed improvement measures has not yet been evaluated. These limitations should be considered when interpreting the findings and planning future quality improvement interventions.

## Conclusions

This clinical audit identified frequent sleep disruption among postoperative paediatric inpatients, with nursing observations and pain emerging as the most commonly reported contributors. Although the overnight ward environment was generally perceived favourably in terms of noise and lighting, opportunities for improvement were identified in the organisation of overnight care and the assessment of individual sleep needs. Parent and carer sleep was also frequently disrupted, highlighting the wider impact of overnight hospital care. The findings support practical measures including routine assessment of sleep needs, clustering of overnight care where clinically appropriate, continued optimisation of pain management, and maintenance of a quiet, low-light environment. A subsequent audit cycle following implementation of these measures will be important to assess whether these changes improve sleep-related outcomes and to support continued quality improvement in postoperative paediatric care.

## Figures and Tables

**Figure 1:**
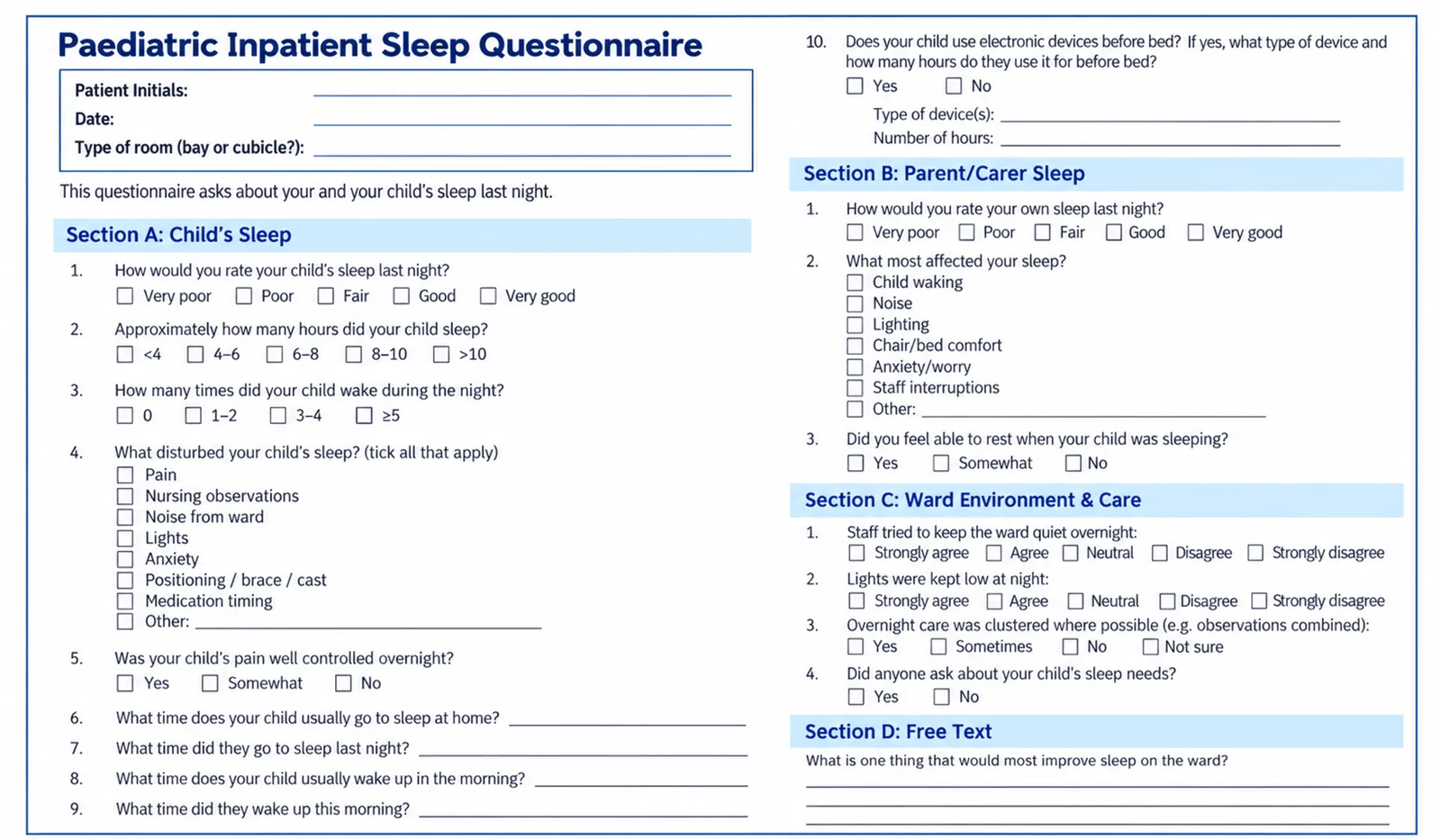
Pediatric inpatient sleep questionnaire template.
